# Physical exercise for bone health in men with prostate cancer receiving androgen deprivation therapy: a systematic review

**DOI:** 10.1007/s00520-020-05830-1

**Published:** 2020-10-29

**Authors:** Barbara Bressi, Maribel Cagliari, Massimiliano Contesini, Elisa Mazzini, Franco Antonio Mario Bergamaschi, Alfredo Moscato, Maria Chiara Bassi, Stefania Costi

**Affiliations:** 1 PhD Program in Clinical and Experimental Medicine, Department of Biomedical, Metabolic and Neural Sciences, University of Modena and Reggio Emilia, Reggio Emilia, Italy; 2 Physical Medicine and Rehabilitation Unit, Azienda USL–IRCCS di Reggio Emilia, Reggio Emilia, Italy; 3 Department of Surgery, Medicine, Dentistry and Morphological Sciences, University of Modena and Reggio Emilia, Modena, Italy; 4 Human Resource Development - Training Radiographers and Radiations Terapist, Azienda USL-IRCCS di Reggio Emilia , Reggio Emilia, Italy; 5 Medical Directorate Hospital Network, Azienda USL-IRCCS di Reggio Emilia , Reggio Emilia, Italy; 6 Urology and Mininvasive Surgery, Department of General and Specialist Surgeries, Azienda USL-IRCCS di Reggio Emilia , Reggio Emilia, Italy; 7 Medical Library, Azienda USL-IRCCS di Reggio Emilia , Reggio Emilia, Italy; 8 Scientific Directorate , Azienda USL-IRCCS di Reggio Emilia , Reggio Emilia, Italy

**Keywords:** Prostatic neoplasms, Exercise, Accidental falls, Fractures, bone, Physical therapy modalities, Prevention

## Abstract

**Purpose:**

Androgen deprivation therapy (ADT) is a treatment used in men with prostate cancer (PCa); however it is responsible for many adverse effects, with negative impact on quality of life. ADT causes loss of bone mineral density (BMD) and skeletal muscle mass, alteration of body composition, and cognitive function, which altogether lead to increased risk of accidental falls and fractures. This systematic review analyses the effectiveness of physical exercise (PE) in preventing accidental falls and fractures and reducing the loss of BMD in men with PCa receiving ADT.

**Methods:**

We searched MEDLINE, EMBASE, CINAHL, and the Cochrane Library for articles between database inception and September 2, 2020. Eligible studies included randomized controlled trials (RCTs) investigating the effects of exercise on bone health in men with PCa receiving ADT.

**Results:**

Nine RCTs were included. Experimental PE consisted in multicomponent programmes that involved aerobic, resistance, impact-loading exercise, and football training. None of the RCTs investigated the risk of accidental falls and fractures, while two trials reported beneficial effects of PE on lumbar spine, hip, and femoral shaft BMD. No further significant difference was detected in the outcomes investigated.

**Conclusion:**

Evidence of the effectiveness of PE to prevent the risk of accidental falls and fractures and BMD loss is lacking. Nevertheless, clinical guidelines recommend PE as a part of the clinical management of men with PCa receiving ADT due to its known numerous health benefits. Research should focus on PE strategies to prevent accidental falls, a clinically relevant outcome in this vulnerable population.

**Trial registration:**

The study protocol was registered with International Prospective Register of Systematic Reviews (PROSPERO, number CRD 42020158444) on 04/28/2020.

**Electronic supplementary material:**

The online version of this article (10.1007/s00520-020-05830-1) contains supplementary material, which is available to authorized users.

## Introduction

Prostate cancer (PCa) is the most prevalent cancer among men worldwide, with 3.724.658 cases in 2018 [1].

Androgen deprivation therapy (ADT) is currently the standard systemic treatment in patients with metastatic or more aggressive PCa [2]. Often, ADT is used in combination with radiotherapy for localized advanced PCa with the aim to increase survival and control disease progression [2].

However, ADT is responsible for many adverse effects, with negative impact on quality of life [3]. Apart from the increased risk for cardiovascular events and metabolic syndrome [4, 5], ADT also alters the body composition, with loss in skeletal muscle mass that leads to a decrease in muscle strength [6, 7]. Also, patients on ADT manifest significant loss of bone mineral density (BMD), which occurs especially within the first year of treatment [8] and is associated with higher osteoporosis rates and risk of fractures [9]. Moreover, ADT seems responsible for cognitive dysfunction, although this finding has not been completely clarified and needs further investigation [10].

It is known that both reduction in muscle strength and cognitive dysfunction are predictors of higher fall rates and hospitalization in older adults [11, 12]. Thus, considering the loss of BMD, altogether these side effects of ADT explain the increased risk of accidental falls and fractures in this population [13].

In elderly adults, physical exercise (PE) has been proposed in different modalities as a strategy to produce several health benefits [14]. Recent guidelines addressing elderly adults recommend multicomponent exercise programmes, including resistance and neuromotor exercises, as a strategy to reduce the risk of accidental falls [14] as PE can prevent osteoporosis and improves body composition, muscle strength, and cognitive function [14]. Moderate-vigorous intensity programmes that include balance exercises seem to be particularly effective to reduce the risk of accidental falls [14].

Initial evidence indicates that in patients with cancer, PE may produce numerous benefits on physical performance, quality of life, and cancer-related fatigue [15]. In patients with PCa receiving ADT, exercise is beneficial to body composition, muscle strength, and physical performance [16–18], while its effects on bone health, cardiometabolic risk, quality of life, and cognitive functions remain uncertain [19, 20].

Patients receiving ADT are more exposed to the risk of accidental falls and fractures [9] due to the side effects of this drugs. As PE is recommended in healthy elderly adults to prevent these risks and also to prevent bone loss, we hypothesized that PE could be effective in preventing accidental falls and fractures even in men with PCa receiving ADT. Furthermore, recent evidence suggests PE as strategy to prevent osteoporosis in men receiving ADT when associated with pharmacological therapy [21]. Thus, we conducted this systematic review to search for evidence of the effectiveness of exercise on bone health in this population. Specifically, we searched for randomized controlled trials that implemented PE programmes to prevent accidental falls and fractures and/or to prevent the loss of BMD, in patients with PCa treated with ADT.

## Methods

This systematic review followed the Preferred Reporting Items for Systematic Reviews and Meta-analyses (PRISMA) guidelines [22]. The study protocol was registered with International Prospective Register of Systematic Reviews (PROSPERO, number CRD 42020158444).

### Search strategy and selection criteria

A systematic review of the literature was performed through sequential, individualized searches in MEDLINE, EMBASE, CINAHL, and the Cochrane Library. We searched for studies published up to September 2, 2020, without filters for study design or language. The search terms and strategies used are reported in online resource 1. Duplicates were removed in EndNote (version X7.5). Also, we performed a manual search in the reference lists of the studies included in this review to find any other relevant citation that may have been missed by the electronic search.

We included randomized controlled trials (RCTs) investigating the effects of supervised or unsupervised exercise on bone health in adult individuals with PCa receiving ADT.

Studies were eligible if the experimental intervention consisted of structured PE programmes compared with standard care or placebo active control. When exercise was associated with dietary supplements, studies were included if the exercise was clearly the predominant part of the experimental intervention. Furthermore, studies were eligible if they investigated the number of accidental falls or fractures that occurred in a specific timeframe or if they reported data on bone density by dual-energy X-ray absorptiometry (DEXA). Studies focusing on generalized advice and education on the benefit of exercise or studies that collected data on pathological (and not accidental) fractures were excluded.

### Data analysis

Two investigators (B.B., M.C.) screened the title and abstract of all the citations retrieved to check their appropriateness related to the purpose of this review. The investigators also retrieved and checked for eligibility the full texts of studies deemed appropriate. Then, two investigators (B.B., S.C.) assessed the eligible studies for their methodological quality according to the Cochrane risk-of-bias tool [23]. In the whole process, any disagreement was resolved by discussion and consensus.

Two investigators (B.B., M.C.) extracted the following data from studies included: authors, year and country, sample size and average age, exclusion criteria, bone outcome measures collected and follow-up duration, general characteristics of the experimental intervention and standard care, drop-out rate. When essential data were missing, the investigators requested them from authors (at least three attempts).

## Results

### Bibliographic search results

The electronic search yielded 304 citations, duplications excluded. One more citation was retrieved through the manual search, for a total of 305. According to the screening of title and abstract, 269 citations were excluded because they did not focus on the topic under investigation.

Thirty-six full texts were reviewed for eligibility, 27 of which were excluded for the following reasons: three conference abstracts and six study protocols referred to published full texts already retrieved [24–31]; four studies did not meet inclusion criteria with respect to the outcome, as one measured only pathological fractures [32], and the others did not report data on bone health [31, 33, 34]; one study did not test a structured physical exercise intervention, focusing instead on patient education [35]; two studies compared different structured physical exercise interventions, without comparison to standard care [36, 37]. Finally, eleven studies were also excluded since they reported insufficient data for analysis [38] or were protocols of ongoing studies [39–48]. We contacted the corresponding authors in order to obtain preliminary results (minimum three attempts), but the ones who replied said they had no data to share yet.

**Fig. 1:**
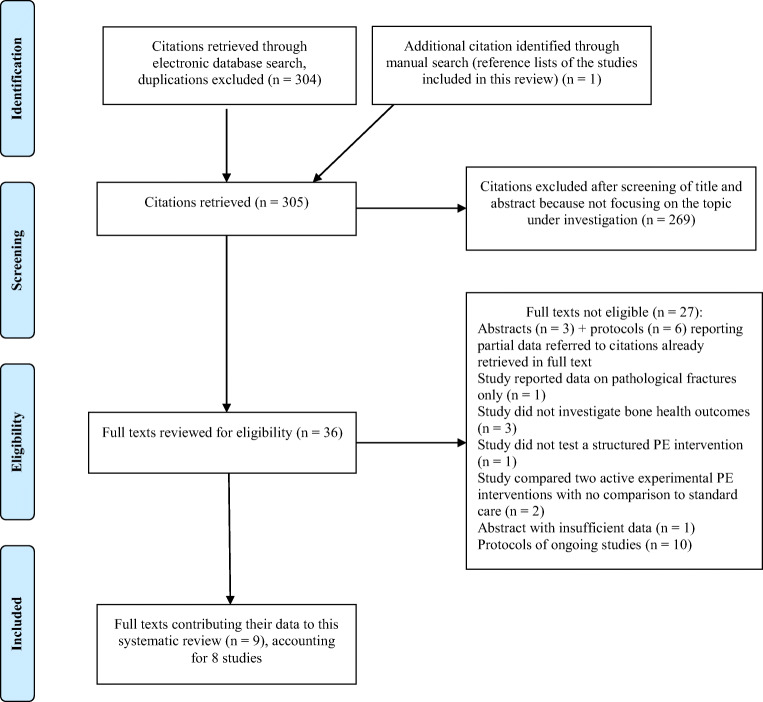
PRISMA flow chart of search and study selection process

Thus, nine published full texts met the inclusion criteria and contributed their data to this review [24–30, 49, 50]. These full texts accounted for eight study designs, as the two by Uth et al. [28, 29] reported data collected at the 3- and 8-month follow-up, respectively, of the same study design and sample (Fig. 1). Of note, the study by Bjerre et al. [30] included patients with PCa regardless of their treatment with ADT. However, they reported specific data for the subgroup of patients on ADT and these data were considered in this review.

### Risk of bias of the included studies

**Fig. 2:**
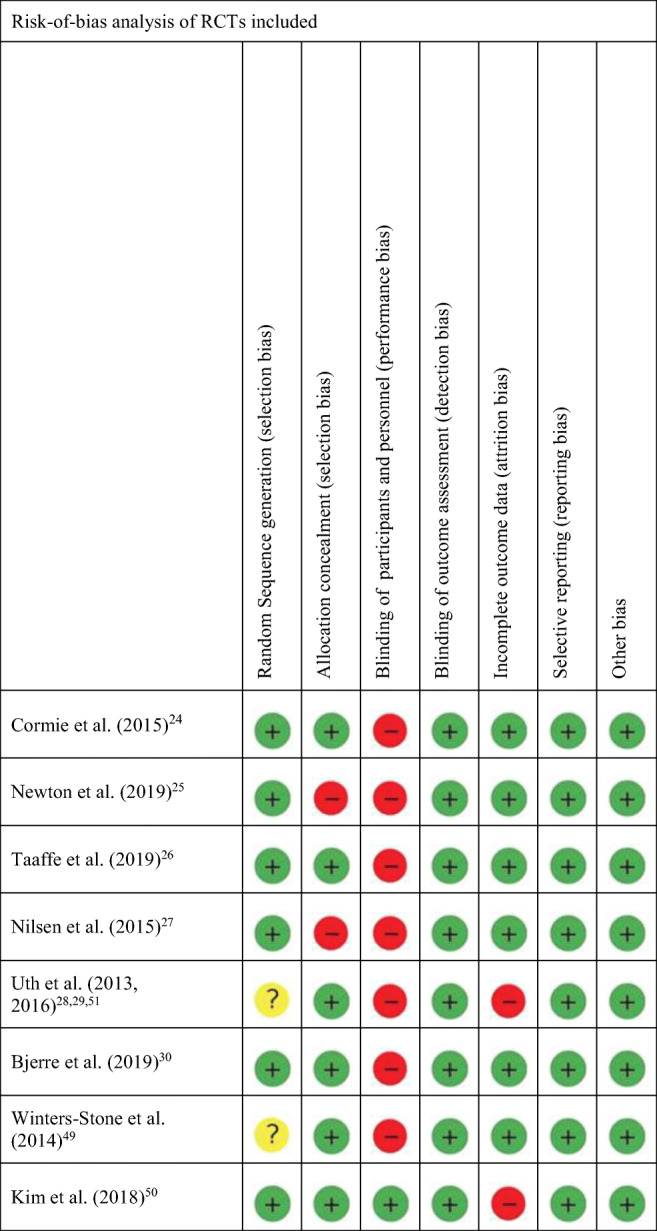
Risk-of-bias analysis of RCTs included

The Cochrane risk-of-bias analysis of the included studies is reported in Fig. 2. Two studies did not report sufficient information to assess the adequacy of the random sequence generation [28, 29, 49, 51]. Due to the nature of the intervention, seven of the nine included studies did not provide blinding to group assignment for both participants and personnel [24–30, 49]. Moreover, four studies did not report enough information to judge blinding of outcome [24–26, 49]. Nevertheless, all the included studies were judged at low risk of detection bias since outcome measures were frequently objective. The analytical assessment of the risk of bias for each study included is reported in online resource 2.

### Study characteristics

Table 1 summarizes the characteristics of the studies included in this review. All were RCTs published in the last decade in different continents [24–30, 49, 50]. All studies allowed for the recruitment of patients treated at several specialized hospitals.

**Table 1:** Characteristics of included studies

First author (year of publication)	Country	Population			Exclusion criteria	Age (mean ± SD years)			Follow-up (months)	Drop-out rate	Bone outcomes
		EG	CG	Tot.		Tot.	EG	CG			
Cormie et al. (2015) [24]	Australia	32	31	63	Restriction to physical exercise based on clinicians’ judgement Musculoskeletal, cardiovascular, neurological disorders Prior exposure to ADT Bone metastasis	68.3*	69.6 (6.5)	67.1 (6.5)	3	13%	aBMD of whole body, lumbar spine (L_2_-L_4_) femoral neck
Newton et al. (2019) [25]	Australia	57 (RE+IE)	47	154	Restriction to physical exercise based on clinicians’ judgement Musculoskeletal, cardiovascular, neurological disorders Current regular resistance exercise Medication that affect bone metabolism Bone metastasis	69.0 (9.0)	68.7 (9.3)	69.1 (8.4)	6	19%	BMD of whole body, total hip, lumbar spine (L_2_-L_4_), femoral neck, trochanter
		50 (AE+RE)					69.1 (9.4)		6	14%	
Taaffe et al. (2019) [26]	Australia	54	50	104	Restriction to physical exercise based on clinicians’ judgement Musculoskeletal, cardiovascular, neurological disorders Current regular resistance or aerobic exercise Prior exposure to ADT Osteoporosis Medication that affect bone metabolism Metastatic disease	68.2*	69.0 (6.3)	67.5 (7.7)	6	18%	BMD of whole body, total hip, lumbar spine
Nilsen et al. (2015) [27]	Norway	28	30	58	Restriction to physical exercise based on clinicians’ judgement Musculoskeletal, cardiovascular, neurological disorders Current regular resistance exercise Medication that affect bone metabolism	66.0	66.0 (6.6)	66.0 (5.0)	4	16%	aBMD of whole body, total hip, total lumbar spine, femoral neck, trochanter
Uth et al. (2016) [28]	Denmark	29	28	57	Musculoskeletal, cardiovascular, neurological disorders Current regular resistance exercise Current chemotherapy Osteoporosis Other cancers	67.0	67.1 (7.1)	66.5 (4.9)	3	14%	aBMD of whole body, legs, total hip, lumbar spine, femoral neck, femoral shaft
Uth et al. (2016) [29]									8	28%	
Bjerre et al. (2019) [30]	Denmark	46	41	87	Restriction to physical exercise based on clinicians’ judgement Osteoporosis	NR	NR	NR	6	NR	BMD of whole body, total hip, lumbar spine, femoral neck
Winters-Stone et al. (2014) [49]	USA	29	22	51	Restriction to physical exercise based on clinicians’ judgement Current regular resistance exercise Current chemotherapy Osteoporosis Medication that affect bone metabolism Bone metastasis	70.2	69.9 (9.3)	70.5 (7.8)	12	29%	BMD of total hip, lumbar spine (L_1_-L_4_), femoral neck, greater trochanter
Kim et al. (2018) [50]	South Korea	26	25	51	Restriction to physical exercise based on clinicians’ judgement Contraindications to unsupervised exercise Current regular resistance exercise Osteoporosis Medication that affect bone metabolism Bone metastasis Other cancers	70.8	70.5 (5.0)	71.0 (5.5)	6	20%	BMD of total hip, lumbar spine (L_1_-L_4_), femoral neck

*aBMD* areal bone mineral density, *ADT* androgen deprivation therapy, *AE* aerobic exercise, *BMD* bone mineral density, *CG* control group, *EG* experimental group, *IE* impact-loading exercise, *NR* not reported for the subgroup that received ADT, *RE* resistance exercise, *SD* standard deviation

*Estimated values

One study was a three-armed RCT comparing two active interventions with one control [25]. For the purposes of this review, we considered both the comparisons. Two studies were cross-over designs, and, for this review, we considered data of the first follow-up, before the cross-over, which was scheduled at 6 months for both studies [25, 26].

A high drop-out rate (≥ 28%) was registered in two studies [29, 49] and equal to 20% in a further one [50], whereas one study did not report this data point for the subgroup of patients receiving ADT [30]. The safety of interventions was assessed by recording the number and type of adverse events. Only two trials reported adverse events related to exercise [27, 28]: one partial Achilles tendon rupture [28], two fibula fractures [28], and five minor musculoskeletal injuries [27, 28]. Other trials registered generic health issues not related to the PE intervention that occurred both in the experimental and in the control group, such as hospitalization (*n* = 22) [25–27, 29, 49, 50], injury/accident (*n* = 10) [25–27], death (*n* = 4) [25, 49], and others (n = 4) [24, 26–29]. Bjerre et al. [30] analysed safety outcomes as number of falls, fractures, and serious adverse events occurring even in the subgroup of patients receiving ADT (respectively *n* = 5, *n* = 1, and *n* = 4), without any further detailed classification.

### Participants

The sample size of the eight RCTs selected in this review ranged from 51 to 154 individuals, for a total of 625 participants, of whom 351 were randomized to receive experimental PE and 274 were randomized to receive standard care. The sample was made up of males aged from 66.0 to 70.8 years with local or metastatic PCa receiving ADT.

All the study designs excluded patients with restrictions to PE based on specific assessments (e.g., inability to walk 400 m [24–26], VO^2^ max < 35 ml/kg/min [28, 29], pain in the metastatic site associated to activity [28, 29]) or based on clinicians’ judgement [24–27, 30, 49, 50]. Five studies also excluded patients with musculoskeletal, cardiovascular, and neurological disorders that could inhibit them from exercising [24–29] or patients with contraindications to unsupervised exercise [50]. Further exclusion criteria have been summarized in detail in Table 1.

### Characteristics of control group intervention and the experimental group intervention

Table 2 summarizes the characteristics of standard care and experimental PE programmes.

**Table 2:** Characteristics of study intervention programmes

First author (year of publication)	Sample	Duration of intervention (months)	EG Component	Experimental PE programme	Frequency (times/weeks)	Session duration (min)	Modality		CG
							Group	Single	
Cormie et al. (2015) [24]	EG: 32 CG: 31	3	AE+RE	Supervised: AE: 20–30 min at 70–85% max HR RE: 6–12 RM, 1–4 sets, reps NR Progression: intensity and volume	2	60	x		Standard care
Newton et al. (2019) [25]	EG: 57 CG: 47	6	RE+IE	Supervised: RE: 6–12 RM, 2–4 sets, reps NR Progression: Intensity and volume IE: 3–5 times BW, 2–4 sets, 10 reps. Progression: Set and type of exercise	2	60	x		Printed booklet with information about exercise
				Unsupervised: IE: two to four rotations of skipping (30 s), hopping, leaping, and drop jumping (all 10 times)	2	60		x	
	EG: 50 CG: 47	6	AE+RE	Supervised: RE: 6–12 RM, 2–4 sets, reps NR Progression: Intensity and volume AE: 20–30 min at 60–85% max HR	2	60	x		
Taaffe et al. (2019) [26]	EG: 54 CG: 50	6	AE+RE+IE	Supervised: AE: 25–40 min of 60–85% max HR RE: 6–12 RM, 2–4 sets, reps NR Progression: intensity and volume IE: 3·4–5·2 times BW, 2–4 sets, 10 reps Progression: set and type of exercise	3	60	x		Standard care
Nilsen et al. (2015) [27]	EG: 28 CG: 30	4	RE	Supervised: RE: 6–10 RM, 1–3 sets, 10 reps. Progression: volume	2	60	x		Encouraged to maintain their habitual physical activity level and not to initiate strength training
				Unsupervised: Home-based exercise session similar to supervised sessions performed at a moderate intensity	1	60	x	x	
Uth et al. (2016) [28]	EG: 29 CG: 28	3	FT	Supervised: 15 min warm-up (drills, balance, strength exercise) 2–3 sets × 15 min Progression: set and frequency	2–3	45–60	x		Encouraged to maintain their habitual physical activity level
Uth et al. (2016) [29]		8							
Bjerre et al. (2019) [30]	EG: 46 CG: 41	6	FT	Supervised: 20 min warm-up 20 min dribbling, passing, and shooting 20 min of 5–7-a-side football Intensity and progression are NR	2	60	x		15/30-min telephone session covering options for physical activity and free-of-charge rehabilitation delivery by the municipalities and subsequently an email with the same information
Winters-Stone et al. (2014) [49]	EG: 29 CG: 22	12	RE+IE	Supervised: RE upper body: 8–15 RM, 1–2 sets, 8–14 reps. RE lower body: 0–15% BW, sets NR, 8–12 reps IE: 0–10% BW, 1–10 sets, 10 reps Progression: Intensity and volume	2	60	x		Performed a series of whole body stretching and relaxation exercises
				Unsupervised: Home-based exercise similar to class session but performed without weighted vests and replacing weights with resistance bands	1	60		x	
Kim et al. (2018) [50]	EG: 26 CG: 25	6	AE+RE	Unsupervised: Core program: Weight-bearing + RE RE: 0–10% BW, 2–3 sets, 8–15 reps. IE: weight-bearing exercise 11–15 RPE, 3–5 sets, 20–30 min Progression: intensity, volume, and type of exercise Optional programme: stabilization/balance exercise + Circuit Resistive Calisthenics (same dose as core program) Stabilization/balance exercise: intensity and volume NR	2–5	NR	NR		Stretching 3–5 times/week (a total of 9 movements); Ten-minute telephone sessions (weekly for the first month and at monthly intervals thereafter)

*AE* aerobic exercise, *BW* body weight, *CG* control group, *EG* experimental group, *FT* football training, *HR* heart rate, *IE* impact-loading exercise, *NR* not reported, *PE* physical exercise, *RE* resistance exercise, *Reps* repetitions, *RM* repetition maximum, *RPE* rate of perceived exertion, *S* seconds

The control group intervention consisted of stretching activities [49, 50] or educational material [25, 30, 50] or simply in encouraging patients to maintain their usual level of physical activity [27–29]. Taaffe et al. [26] provided all participants with standard daily supplementation of calcium (1000 mg/day) and vitamin D3 (800 IU/day).

Experimental PE interventions were characterized by multicomponent programmes [24–26, 28–30, 49, 50], with only one exception that implemented a single component of PE [27]. In most cases, PE consisted in aerobic exercise (AE) that could also be performed as weight-bearing activities and that was associated with resistance exercise (RE) [24–26, 50] and with impact-loading exercise (IE) [26]. Two study designs implemented football training (FT) as experimental PE [28–30]; although the full texts did not report this type of training in detail, it is likely that, by its nature, it included AE and IE, among others (e.g., RE, stretching).

Most experimental interventions were performed in 1-hour sessions repeated two or three times a week [24–30, 49]. Most of the studies described how the PE components were progressively modulated in terms of intensity, volume, and type of exercise [24–27, 49, 50]. Although the intensity of FT was not defined, this type of intervention was implemented through an initial warm-up followed by 2 matches lasting 15/20 min [28–30]. Uth et al. [28, 29] progressively increased the number of matches and the frequency of sessions per week.

Experimental PE were implemented as supervised exercise in clinics [24–26] or in sports facilities [28–30], as a combination of supervised and unsupervised (home-based) sessions [25, 27, 49], or as unsupervised home-based PE only [50].

### Bone outcomes

#### Accidental falls and fractures

None of the studies selected for this review was designed to analyse the risk of accidental falls and/or fractures as an outcome measure of the effectiveness of experimental PE in reducing those risks. All study designs measured adverse events [24–30, 49, 50]. The two studies that tested FT also recorded fractures occurring during this kind of intervention to judge its safety due to the increased risk of collisions with other players and falls [28–30]. Although this was not the outcome we were interested in, we underline that all the experimented PE programmes were deemed safe [24–30, 49, 50].

Of note, several studies secondarily collected data on physical function through heterogeneous tests (e.g., Flamingo balance test, sit-to-stand test, etc.) [24, 27–29, 50]. The proof of effectiveness of experimental PE was demonstrated through the sit-to-stand test [24, 27, 50], which is valid to measure muscle power of the lower limbs; its validity in predicting accidental falls and fractures in patients with cancer, however, must still be demonstrated [52].

#### BMD

Table 3 reports the results of between-group comparisons of BMD at the various anatomical sites.

**Table 3:** Between-group comparisons for BMD

	Follow-up (months)	BMD outcome	Mean change between groups (g/cm^2^)	95% CI	*p* value
Cormie et al. (2015) [24]	3	Whole body	− 0.002*	− 0.013 to 0.009	0.692
		Lumbar spine	− 0.009*	− 0.029 to 0.012	0.410
		Femoral neck	0.000*	− 0.025 to 0.024	0.987
Newton et al. (2019) [25]	6 (RE+IE)	Whole body	0.005*	− 0.002 to 0.011	0.174
		Total hip	0.007*	− 0.002 to 0.016	0.128
		Lumbar spine	0.014*	0.001 to 0.027	0.039
		Femoral neck	0.010*	0.000 to 0.020	0.050
		Trochanter	− 0.003*	− 0.010 to 0.004	0.449
	6 (AE+RE)	Whole body	0.003*	− 0.007 to 0.0012	0.614
		Total hip	0.001*	− 0.009 to 0.011	0.807
		Lumbar spine	0.004*	− 0.009 to 0.017	0.525
		Femoral neck	− 0.003*	− 0.014 to 0.008	0.571
		Trochanter	− 0.002*	− 0.01 to 0.007	0.699
Taaffe et al. (2019) [26]	6	Whole body	NR	NR	0.827
		Total hip	NR	NR	0.848
		Lumbar spine	NR	NR	0.111
Nilsen et al. (2015) [27]	4	Whole body	0.00*	− 0.02 to 0.01	0.520
		Total hip	0.00*	− 0.01 to 0.01	0.690
		Total lumbar spine	0.00*	− 0.02 to 0.01	0.847
		Femoral neck	0.00*	− 0.02 to 0.01	0.467
		Trochanter	0.00*	− 0.01 to 0.00	0.221
Uth et al. (2016) [28]	3	Whole body	0.01	− 0.00 to 0.01	0.188
		Legs	0.00	− 0.00 to 0.01	0.336
Uth et al. (2016) [29]	8	Total hip	R: 0.015	0.003 to 0.027	0.015
			L: 0.017	0.002 to 0.032	0.030
		Lumbar spine	0.028	− 0.010 to 0.065	0.144
		Femoral neck	R: 0.015	− 0.002 to 0.031	0.078
			L: 0.015	− 0.01 to 0.032	0.072
		Femoral shaft	R: 0.018	0.004 to 0.032	0.016
			L: 0.024	0·005 to 0·044	0.015
Bjerre et al. (2019) [30]	6	Whole body	0.005	− 0.007 to 0.017	0.40
		Total hip	− 0.009*	− 0.033 to 0.014	0.43
		Lumbar spine	0.0017*	− 0.019 to 0.053	0.34
		Femoral neck	0.007*	− 0.009 to 0.023	0.39
Winters-Stone et al. (2014) [49]	12	Total hip	NR	NR	0.37
		Lumbar spine	NR	NR	0.47
		Femoral neck	NR	NR	0.77
		Greater trochanter	NR	NR	0.58
Kim et al. (2018) [50]	6	Total hip	NR	NR	0.727
		Lumbar spine	NR	NR	0.756
		Femoral neck	NR	NR	0.888

*AE* aerobic exercise, *BMD* bone mineral density, *CI* confidence intervals, *IE* impact-loading exercise, *L* left, *NR* not reported, *R* right, *RE* resistance exercise

*Analyses adjusted for baseline values

All the RCTs selected for this review reported data on bone density measured by DEXA at different anatomical sites (Table 3). Lumbar spine BMD was collected in all the included studies, while femoral neck BMD was analysed in seven of them [24, 25, 27, 29, 30, 49, 50]. At the 6-month follow-up, Newton et al. [25] recorded a significant difference between groups for lumbar spine BMD (mean change 0.014 g/cm^2^, 95% CI 0.001–0.027, *p* = 0.039) and a positive trend for femoral neck BMD (mean change 0.010 g/cm^2^, 95% CI 0.000–0.020, *p* = 0.050), in favour of the experimental resistance and impact-loading PE compared with control group. No further significant difference was detected by any of the studies in these outcome measures. Of note, a per-protocol analysis performed by Winters-Stone et al. [49] at the level of single lumbar vertebra reported a significant difference in BMD only for L_4_ (*p* = 0.03), in favour of experimental PE.

Total hip BMD was measured by seven study designs [25–27, 29, 30, 49, 50], with significant differences recorded only by Uth et al. [29] on both hips at the 8-month follow-up (right 0.015 g/cm^2^, 95% CI 0.003–0.027, *p* = 0.015; left 0.017 g/cm^2^, 95% CI 0.002–0.032, *p* = 0.030). This study was the only one that collected data on BMD at the femoral shaft of both legs, recording a difference in favour of experimental PE on both sides (right 0.018 g/cm^2^, 95% CI 0.004–0.032, *p* = 0.016; left 0.024 g/cm^2^, 95% CI 0.005–0.044, *p* = 0.015) [29].

No further statistically significant difference was registered for BMD at any further anatomical site examined, such as whole body [24–28, 30], trochanter [25, 27, 49], or legs [28].

Considering the almost total absence of data in favour of PE with respect to this outcome, which was collected in various anatomical sites, we deemed it inappropriate to carry out a meta-analysis.

### Further results: bone turnover markers

Six study designs also assessed several bone turnover markers (BTMs) such as markers of bone formation (alkaline phosphatase, procollagen type 1 amino-terminal propeptide and osteocalcin) or markers of bone resorption (C-terminal telopeptide of type I collagen, N-terminal telopeptide of type I collagen) [24–26, 28, 29, 49, 50].

At the 3-month follow-up, Uth et al. [28, 29] registered a statistically significant difference in favour of experimental PE for the markers of bone formation procollagen type 1 amino-terminal propeptide (36.6 μg/L, 95% CI 10.4–62.8, *p* = 0.008) and osteocalcin (8.6 μg/L, 95% CI 3.3–13.8, *p* = 0.002), but this result was not confirmed at the subsequent 8-month follow-up.

## Discussion

The aim of this systematic review was to summarize the evidence regarding the effectiveness of PE programmes in reducing the risk of accidental falls and fractures, as a clinically relevant outcome in the PCa patient population.

Despite the strong existing evidence proving accelerated bone loss, additional muscle weakness, and cognitive dysfunction caused by ADT [7, 10] and the benefits of PE in reducing the risk of accidental falls in the healthy elderly population [14], no study has investigated the effects of exercise on these clinically relevant endpoints in PCa patients.

Furthermore, the effectiveness of PE in lessening or preventing the loss of BMD in this population is still uncertain due to the inconsistent results yielded by this systematic review. In particular, only two studies suggested that multicomponent PE, in particular resistance and impact-loading exercise or football training, may help achieve this outcome [25, 29]. These positive results could be explained by the longer period of training [25], as a minimum of 6–8 months is required to achieve bone remodelling [53], or by the high number of accelerations and decelerations and change of direction typically observed during the football training [29]. These characteristics of the PE intervention could provide sufficient bone-loading forces and osteogenic stimulus. However, both these study designs were affected by a certain degree of risk of bias. Therefore, although BMD was measured objectively, their results should be interpreted with caution.

Thus, to date, evidence of the beneficial effects of PE on bone health in men with PCa treated with ADT is still lacking, even though PE is beneficial in the healthy elderly for the same outcome [14].

The incidence of osteoporotic fractures increases with age, and it is estimated that more than 8.9 million osteoporotic fractures occur annually worldwide [54, 55]. In men over the age of 75, the most frequent site of fracture is the hip, the principal risk factor being low BMD [56]. Moreover, more than 30% of community-dwelling older adults over the age of 75 fall every year [57], leading to fractures, hospitalizations, and admission to nursing homes [58].

Therefore, the risk of falls in older adults increases morbidity, mortality, and financial burden for societies [59]. Indeed, the costs associated with fragility fractures account for €37 billion/year in Europe and are expected to increase [54], whereas in the USA, the overall number of healthy years of life lost (DALY) due to hip fractures is roughly 17,660 [60].

Considering the progressive aging of the population, recent guidelines suggest that future research should identify individuals at increased risk of fracture, to whom fracture prevention strategies should be targeted [61] in order to contain the increase in costs associated with this event [62]. We think that patients with PCa receiving ADT are among those individuals because the side effects they have affect bone and lead to double the healthcare cost per person [63]. Moreover, as fall prevention programmes are recommended to the elderly in general, as community-dwelling adults with cancer have greater accidental fall rates than do healthy elderly individuals and as patients undergoing active treatment are even more at risk [64], it seems logical to expect that the beneficial effects of PE would be greater in patients with PCa receiving ADT compared with healthy elderly individuals.

However, this systematic review demonstrated an almost complete lack of studies supporting this. Not only have clinically relevant outcomes never been investigated but also few of the RCTs included were powered to detect the effects of PE on BMD [25, 26, 49, 50].

We must say, however, that our review was limited to collecting evidence on PE. As we did not consider other types of interventions, such as nutritional and educational programmes that could help to prevent accidental falls and fractures, we cannot rule out that an evidence-based intervention different from stand-alone PE could be successfully applied. Furthermore, ten protocols of ongoing studies were retrieved by our search strategy; it is therefore very likely that in the next few years the conclusion drawn today, thanks to this extensive review conducted with rigorous methodology, will be outdated.

A final consideration regards the type of PE programmes tested in the studies included in this review: most combined different exercise modalities, such as resistance, weight-bearing endurance, and impact loading exercises, as recommended to provide benefits to bone health [53]. Moreover, PE programmes were of moderate-high intensity in all cases, suggesting that, according to the evidence and expertise, low-impact exercise may have no effect on BMD [53]. Despite this, the PE programmes did not produce the desired result on bone mass. This may be due to the insufficient power of some of the included studies, or it may be due to poor adherence to treatment, which is always an issue in studies involving lifestyle changes [65]. Poor adherence to treatment means that the expected dose of exercise is not achieved by participants. Thus, it can be difficult to determine the effect of the exercise on the outcomes of interest. It could also be that, however intense the programme, PE may not be sufficient to counteract the loss of bone mass induced by aging and ADT.

Thus, to conclude, experts recommend exercise as part of the treatment regimen of patients with cancer thanks to its large number of health benefits [19]. This review suggests that there is still no strong evidence to support this choice to prevent bone density loss in patients with PCa receiving ADT, according to recent literature [16, 21]. However, since exercise is an effective strategy to produce a large number of health benefits, future research should investigate the effects of PE to prevent the risk of accidental falls in this population, which is a clinically relevant outcome. For this purpose, PE should include coordination and balance exercises as well as muscle-strengthening activities. Evidence is needed regarding more precise training components, dose, and progression of exercise to prevent falls.

## Electronic supplementary material

ESM 1 (DOCX 18 kb)

ESM 2 (DOCX 32 kb)
