# Supplementary material for: Physical exercise for bone health in men with prostate cancer receiving androgen deprivation therapy: a systematic review
Source: Support Care Cancer. 2020 Oct 29;29(4):1811–24. doi: 10.1007/s00520-020-05830-1 (PMC7892525; doi:10.1007/s00520-020-05830-1)
Supplement: Supplementary file 1 — (DOCX 18 kb) [file 520_2020_5830_MOESM1_ESM.docx]

**Title:** Physical Exercise for Bone Health in Men with Prostate Cancer Receiving Androgen Deprivation Therapy: a Systematic Review

**Journal:** Supportive Care in cancer

**Author names**: Barbara Bressi^1,2^**,** Maribel Cagliari^3^, Massimiliano Contesini^2^, Elisa Mazzini^2^, Franco Antonio Mario Bergamaschi^2^, Alfredo Moscato^2^, Maria Chiara Bassi^2^, Stefania Costi^2,3^

**Author Affiliations:**

1. PhD Program in Clinical and Experimental Medicine, Department of Biomedical, Metabolic and Neural Sciences, University of Modena and Reggio Emilia, Reggio Emilia, Italy.
2. Azienda USL–IRCCS di Reggio Emilia. Reggio Emilia, Italy.
3. Department of Surgery, Medicine, Dentistry and Morphological Sciences, University of Modena and Reggio Emilia, Modena, Italy.

**E-mail of the corresponding author:** barbara.bressi@ausl.re.it

**Appendix 1 -** Research strategies

**Ovid Medline on 2/07/2020**

#1 "Androgen Antagonists"[Mesh] OR "Gonadotropin-Releasing Hormone"[Mesh]

#2 hormone* OR androgen OR androgen deprivation therapy

#3 #1 OR #2

#4 "Prostatic Neoplasms"[Mesh]

#5 prostat* AND (cancer OR tumor OR neoplasm)

#6 #4 OR #5

#7 "Exercise"[Mesh]

#8 "Physical Therapy Modalities"[Mesh]

#9 exercise* OR physical activit* OR physical therap*

#10 #7 OR #8 OR #9

#11 #3 AND #6 AND #10

**Embase on 2/07/2020**

#1. hormone*:ab,ti OR androgen:ab,ti OR 'androgen deprivation therapy':ab,ti

#2. 'antiandrogen'/exp/mj OR 'gonadorelin derivative'/exp/mj

#3. #1 OR #2

#4. prostat*:ab,ti AND (cancer:ab,ti OR tumor:ab,ti OR neoplasm:ab,ti)

#5. 'prostate tumor'/exp/mj

#6. #4 OR #5

#7. exercise*:ab,ti OR 'physical activit*':ab,ti OR 'physical therap*':ab,ti

#8. 'exercise'/exp/mj OR 'physiotherapy'/exp/mj

#9. #7 OR #8

#10. #3 AND #6 AND #9

**CINAHL on 2/07/2020**

S1 hormone* OR androgen OR androgen deprivation therapy

S2 (MH "Androgen Antagonists")

S3 (MH "Gonadorelin")

S4 prostat* AND (cancer OR tumor OR neoplasm)

S5 (MH "Prostatic Neoplasms")

S6 (MH "Exercise")

S7 (MH "Physical Therapy")

S8 exercise* OR physical activit* OR physical therap*

S9 S1 OR S2 OR S3

S10 S4 OR S5

S11 S6 OR S7 OR S8

S12 S9 AND S10 AND S11

**Cochrane Library on 2/07/2020**

#1 MeSH descriptor: [Androgen Antagonists] explode all trees

#2 MeSH descriptor: [Gonadotropin-Releasing Hormone] explode all trees

#3 (hormone* OR androgen OR androgen deprivation therapy):ti,ab,kw

#4 #1 or #2 or #3

#5 MeSH descriptor: [Prostatic Neoplasms] explode all trees

#6 (prostat* AND (cancer OR tumor OR neoplasm)):ti,ab,kw

#7 #5 or #6

#8 MeSH descriptor: [Exercise] explode all trees

#9 MeSH descriptor: [Physical Therapy Modalities] explode all trees

#10 (exercise* OR physical activit* OR physical therap*):ti,ab,kw

#11 #8 or #9 or #10

#12 #4 and #7 and #11
