# Supplementary material for: Physical exercise for bone health in men with prostate cancer receiving androgen deprivation therapy: a systematic review
Source: Support Care Cancer. 2020 Oct 29;29(4):1811–24. doi: 10.1007/s00520-020-05830-1 (PMC7892525; doi:10.1007/s00520-020-05830-1)
Supplement: Supplementary file 2 — (DOCX 32 kb) [file 520_2020_5830_MOESM2_ESM.docx]

**Title:** Physical Exercise for Bone Health in Men with Prostate Cancer Receiving Androgen Deprivation Therapy: a Systematic Review

**Journal:** Supportive Care in cancer

**Author names**: Barbara Bressi^1,2^**,** Maribel Cagliari^3^, Massimiliano Contesini^2^, Elisa Mazzini^2^, Franco Antonio Mario Bergamaschi^2^, Alfredo Moscato^2^, Maria Chiara Bassi^2^, Stefania Costi^2,3^

**Author Affiliations:**

1. PhD Program in Clinical and Experimental Medicine, Department of Biomedical, Metabolic and Neural Sciences, University of Modena and Reggio Emilia, Reggio Emilia, Italy.
2. Azienda USL–IRCCS di Reggio Emilia. Reggio Emilia, Italy.
3. Department of Surgery, Medicine, Dentistry and Morphological Sciences, University of Modena and Reggio Emilia, Modena, Italy.

**E-mail of the corresponding author:** barbara.bressi@ausl.re.it

| **Appendix 2** The analytical assessment of the risk of bias for each study included | | |
| --- | --- | --- |
| **Item** | **Authors’ judgement** | **Description** |
| **Cormie et al (2015)^24^** | | |
| Sequence generation | Low risk | Quote: “Participants were randomised in an allocation ratio of 1:1 using a random assignment computer program”. |
| Allocation concealment | Low risk | Quote: “The project coordinator and the exercise physiologists involved in assigning participants to groups were ‘blinded’ to the allocation sequence” |
| Blinding of participants and personnel | High risk | The authors do not specify this aspect.  Comment: probably not done. |
| Blinding of outcome assessment | Low risk | The authors do not specify this aspect.  Comment: outcomes considered in this study and extracted for our review (BMD) are not likely to be influenced by lack of blinding. |
| Incomplete outcome data | Low risk | Quote: “An intention-to-treat approach was used for all analyses using maximum likelihood imputation of missing values (expectation maximization)” |
| Selective reporting | Low risk | All prespecified outcomes were reported. |
| Other bias | Low risk | Study appears to be free of other sources of bias. |
| **Newton et al (2019)^25^** | | |
| Sequence generation | Low risk | Quote: “patients were randomly allocated by computer random assignment”. |
| Allocation concealment | High risk | The authors do not specify this aspect.  Comment: probably not done. |
| Blinding of participants and personnel | High risk | The authors do not specify this aspect.  Comment: probably not done. |
| Blinding of outcome assessment | Low risk | The authors do not specify this aspect.  Comment: outcomes considered in this study and extracted for our review (BMD) are not likely to be influenced by lack of blinding. |
| Incomplete outcome data | Low risk | Quote: “An intention-to-treat approach was used for all analyzes using maximum likelihood imputation of missing values (expectation maximization)”. |
| Selective reporting | Low risk | All pre-specified outcomes were reported. |
| Other bias | Low risk | Study appears to be free of other sources of bias. |
| **Taaffe et al (2019)^26^** | | |
| Sequence generation | Low risk | Quote: “104 men were randomly assigned using a computer random assignment program”. |
| Allocation concealment | Low risk | Quote: “This was a single-blinded randomized controlled trail (RCT; investigators and testing personnel blinded to group allocation)”. |
| Blinding of participants and personnel | High risk | The authors do not specify this aspect.  Comment: probably not done. |
| Blinding of outcome assessment | Low risk | The authors do not specify this aspect.  Comment: outcomes considered in this study and extracted for our review (BMD) are not likely to be influenced by lack of blinding. |
| Incomplete outcome data | Low risk | Quote: “intention-to-treat was used for analyses of primary and secondary endpoints using maximum-likelihood imputation of missing values (expectation maximization)”. |
| Selective reporting | Low risk | All pre-specified outcomes were reported. |
| Other bias | Low risk | The study appears to be free of other sources of bias. |
| **Nilsen et al (2015)^27^** | | |
| Sequence generation | Low risk | Quote: “randomization was computerized in a 1:1 ratio by the staff at the clinical research office at Oslo University Hospital”. |
| Allocation concealment | High risk | The authors do not specify this aspect.  Comment: probably not done. |
| Blinding of participants and personnel | High risk | The authors do not specify this aspect.  Comment: probably not done. |
| Blinding of outcome assessment | Low risk | Quote: “personnel performing DXA scans were blinded to group allocation”. |
| Incomplete outcome data | Low risk | Quote: “missing data were imputed by an intention-to-treat approach using the last observation carried forward”. |
| Selective reporting | Low risk | All prespecified outcomes were reported. |
| Other bias | Low risk | The study appears to be free of other sources of bias. |
| **Uth et al (2016, 2013)^28,29,51^** | | |
| Sequence generation | Unclear | Quote: “after successful completion of all baseline assessments participants are randomized 1:1 to the soccer intervention or control group”.  Comment: insufficient to be confident that the allocation sequence was genuinely randomized. |
| Allocation concealment | Low risk | Quote: “The randomization process will be conducted by a research consultant at the Copenhagen Trial Unit who has no other involvement in the study”. |
| Blinding of participants and personnel | High risk | Quote: “blinding of patients and soccer instructors in this kind of study is not possible”.  Comment: not done. |
| Blinding of outcome assessment | Low risk | Quote: “at the termination of the study a statistician blinded to treatment assignment will perform all analyses before disclosing any study outcome data to the study coordinator and researchers involved in the study”. |
| Incomplete outcome data | High risk | Quote: “Change scores are calculated only on data from participants assessed at both baseline and at 12 weeks”.  Comment: all analyses were conducted per protocol. |
| Selective reporting | Low risk | All pre-specified outcomes were reported. |
| Other bias | Low risk | The study appears to be free of other sources of bias. |
| **Bjerre et al (2019)^30^** | | |
| Sequence generation | Low risk | Quote: “Patients were randomly allocated in two groups according to a computer-generated list of a number” Comment: insufficient to be confident that the allocation sequence was genuinely randomized. |
| Allocation concealment | Low risk | Quote: “The allocation was concealed from trial personnel as the statistician received a password-protected email from the trial management system (easyTrial®) with an upload function for the allocation sequence”. |
| Blinding of participants and personnel | High risk | Quote: “Given the nature of the intervention, neither participants nor coaches were blinded”  Comment: not done. |
| Blinding of outcome assessment | Low risk | Quote: “blinding was implemented for objective outcome, so personnel performing assessment had no information on the group allocation” |
| Incomplete outcome data | Low risk | Quote: “The analyses were performed as described for the ITT population”. |
| Selective reporting | Low risk | All prespecified outcomes were reported. |
| Other bias | Low risk | Study appears to be free of other sources of bias. |
| **Winters-Stone et al (2014)^49^** | | |
| Sequence generation | Unclear | Quote: “we conducted a 12-month single-blind randomized controlled trial comparing two parallel groups”.  Comment: insufficient to be confident that the allocation sequence was genuinely randomized. |
| Allocation concealment | Low risk | Quote: “trained technicians blinded to group assignment”. |
| Blinding of participants and personnel | High risk | The authors do not specify this aspect.  Comment: probably not done. |
| Blinding of outcome assessment | Low risk | The authors do not specify this aspect  Comment: outcomes considered in this study and extracted for our review (BMD) are not likely to be influenced by lack of blinding |
| Incomplete outcome data | Low risk | Quote: “data were initially analyzed using an intention-to-treat (ITT) approach via Hierarchical Linear Modeling keeping each participant within his originally assigned group and regardless of missing data”. |
| Selective reporting | Low risk | All prespecified outcomes were reported |
| Other bias | Low risk | The study appears to be free of other sources of bias |
| **Kim et al (2018)^50^** | | |
| Sequence generation | Low risk | Quote: “A block randomization (block size 4) procedure, using computer-generated randomization numbers”. |
| Allocation concealment | Low risk | Quote: “group assignments were placed in sealed, sequentially numbered envelopes and opened by the participants”. |
| Blinding of participants and personnel | Low risk | Quote: “group assignments were placed in sealed, sequentially numbered envelopes and opened by the participants, who were blind to group assignment (they were informed only that they would be given 1 of 2 types of exercise)”. |
| Blinding of outcome assessment | Low risk | Quote: “licensed technicians blind to study groups measured BMD by dual-energy absorptiometry using…”. |
| Incomplete outcome data | High risk | Quote: “All analyses were conducted per protocol”. |
| Selective reporting | Low risk | All prespecified outcomes were reported. |
| Other bias | Low risk | Study appears to be free of other sources of bias. |
